# Association Between Phenotypic Age and the Risk of Mortality in Patients With Heart Failure: A Retrospective Cohort Study

**DOI:** 10.1002/clc.24321

**Published:** 2024-08-08

**Authors:** Xuhong Xu, Zhiqi Xu

**Affiliations:** ^1^ Department of Cardiovascular Medicine Huadu District People's Hospital of Guangzhou Guangzhou Guangdong People's Republic of China

**Keywords:** all‐cause mortality, heart failure, phenotypic age, phenotypic age acceleration

## Abstract

**Background:**

Chronological age (CA) is an imperfect proxy for the true biological aging state of the body. As novel measures of biological aging, Phenotypic age (PhenoAge) and Phenotypic age acceleration (PhenoAgeAccel), have been shown to identify morbidity and mortality risks in the general population.

**Hypothesis:**

PhenoAge and PhenoAgeAccel might be associated with mortality in heart failure (HF) patients.

**Methods:**

This cohort study extracted adult data from the National Health and Nutrition Examination Survey (NHANES) databases. Weighted univariable and multivariable Cox models were performed to analyze the effect of PhenoAge and PhenoAgeAccel on all‐cause mortality in HF patients, and hazard ratio (HR) with 95% confidence intervals (CI) was calculated.

**Results:**

In total, 845 HF patients were identified, with 626 all‐cause mortality patients. The findings suggested that (1) each 1‐ and 10‐year increase in PhenoAge were associated with a 3% (HR = 1.03, 95% CI: 1.03–1.04) and 41% (HR = 1.41, 95% CI: 1.29–1.54) increased risk of all‐cause mortality, respectively; (2) when the PhenoAgeAccel < 0 as reference, the ≥ 0 group was associated with higher risk of all‐cause mortality (HR = 1.91, 95% CI = 1.49–2.45). Subgroup analyses showed that (1) older PhenoAge was associated with an increased risk of all‐cause mortality in all subgroups; (2) when the PhenoAgeAccel < 0 as a reference, PhenoAgeAccel ≥ 0 was associated with a higher risk of all‐cause mortality in all subgroups.

**Conclusion:**

Older PhenoAge was associated with an increased risk of all‐cause mortality in HF patients. PhenoAge and PhenoAgeAccel can be used as convenient tools to facilitate the identification of at‐risk individuals with HF and the evaluation of intervention efficacy.

## 1 Introduction

Heart failure (HF) is a typical cardiovascular syndrome of aging, has become a public health problem, with considerable healthcare and economic burden [1, 2, 3]. The HF‐attributable mortality rate has increased by over 20% since 2011, and the median survival time after an initial HF diagnosis is as low as 2.1 years [4, 5]. Despite many advances in understanding its pathophysiology and associated targeted therapeutic strategies, mortality remains unacceptably high [5]. Finding indicators that are closely associated with the risk of mortality in HF patients is critical to identifying individuals at high risk for poor prognosis.

Biological aging is a progressive decline in homeostasis that is associated with prognosis in patients with HF [6, 7]. Given that aging rate varies among individuals, chronological age (CA) is not an appropriate indicator for the true biological aging state of the body [8]. Phenotypic age (PhenoAge) is a novel measure of biologic aging, that is highly predictive of mortality even in different stratification, such as by age, disease count, and health behavior [9]. Phenotypic age acceleration (PhenoAgeAccel), represents PhenoAge after accounting for CA (a positive value means the person appears older than expected) indicates biological aging [9, 10]. Studies have shown that PhenoAge and PhenoAgeAccel can capture different subgroups of morbidity and mortality risk, including healthy and unhealthy individuals and patients with heart disease in different age groups [11, 12]. In addition, high PhenoAge/PhenoAgeAccel was associated with a high risk of all‐cause and cardiovascular disease (CVD) mortality in patients with multivessel coronary artery disease and diabetes [13, 14]. To the best of our knowledge, however, the association of PhenoAge and PhenoAgeAccel with the risk of all‐cause mortality and CVD mortality in HF patients remains unclear.

Herein, we investigated the association of PhenoAge and PhenoAgeAccel with risk of all‐cause mortality and CVD mortality in HF patients, and further explored whether this association remains in patients stratified by gender, stroke, diabetes, dyslipidemia, and chronic kidney disease (CKD), which may provide certain reference for identifying high‐risk HF patients with poor prognosis.

## 2 Methods

### 2.1 Study Design and Population

Data of HF patients in this retrospective cohort study was extracted from the National Health and Nutrition Examination Survey (NHANES) databases (1999–2010), which was a major program of the National Center for Health Statistics (NCHS) that combines interviews and physical examinations to assess the health and nutritional status of adults and children in the United States [15]. Institutional review board approval was not required for this study because NHANES database is a deidentified and publicly available (https://www.cdc.gov/nchs/nhanes/index.htm). The requirement of ethical approval for this was waived by the Institutional Review Board of Huadu District People's Hospital of Guangzhou, because the data was accessed from NHANES (a publicly available database). The need for written informed consent was waived by the Institutional Review Board of Huadu District People's Hospital of Guangzhou due to retrospective nature of the study. All methods were performed in accordance with the relevant guidelines and regulations.

Inclusion criteria were as follows: (1) patients aged ≥ 18 years; (2) patients diagnosed with HF [12, 16]. Exclusion criteria were as follows: (1) patients with missing PhenoAge parameters; (2) patients with missing data of HF diagnosis age, education level, and marital status; (3) patients with missing survival data.

### 2.2 Phenotypic Age and Phenotypic Age Acceleration

PhenoAge was determined using CA and nine clinical biomarkers (red blood cell distribution width [RDW], albumin, lymphocyte percent, mean cell volume, white blood cell [WBC] count, creatinine, glucose, alkaline phosphatase, and [log] C‐reactive protein [CRP]) [17]. The equation for calculating PhenoAge was based on two parametric proportional hazards models—one fit using all 10 selected variables and the other fit using only CA. The calculation equation is as follows:

$\mathrm{Phenotypic}\;\mathrm{age}=141.50+\frac{\ln[-0.00553\times \ln(1-M)}{0.09165},$

where

$$M=1-\text{exp}(\frac{-1.51714\times \text{exp}(\mathrm{xb})}{0.0076927}),$$

and xb=−19.907−0.0336×Albumin+0.0095×Creatinine+0.1953×Glucose+0.0954×ln(CRP)−0.0120×Lymphocyte percent+0.0268×Mean cell volume+0.3306×RDW+0.00188×Alkaline  phosphatase+0.0554×WBCcount+0.0804×Chronological age.

In addition, the PhenoAgeAccel was calculated according to the residual resulting by the linear model in the regression of PhenoAge on the CA. Therefore, PhenoAgeAccel represents PhenoAge after accounting for CA (i.e., whether a person's biological age [BA] is older [≥ 0] or younger [< 0] than expected based on his/her CA).

### 2.3 Potential Covariates

Potential covariates included age, gender (female/male), race (non‐Hispanic White, other Hispanic, Mexican American, non‐Hispanic Black, or other), education level (< 9th grade, 9–11th grade, high school grad/GED/equivalent, some college/AA degree, or college graduate/higher), family income ratio (PIR) (< 1, ≥ 1, unknown), marital status (married or not married), energy intake, overweight, hemoglobin and uric acid. Two smoking status categories were created, which included smokers (≥ 100 cigarettes during one's lifetime) and never‐smokers (< 100 cigarettes during one's lifetime). Alcohol consumption (drinking at least 12 drinks in lifetime/per year or not). Drinking status was classified as drinker (had ≥ 12 drinks in 1 year or drank ≥ 12 drinks in life), never drinker (had < 12 drinks in 1 year or had < 12 drinks in life). Physical activity level was classified as high level (any one of the vigorous work activity and vigorous recreational activities, or any two of moderate work activity and walk or bicycle and moderate recreational activities, or any one of walked or bicycled over past 30 days and vigorous activity over past 30 days and moderate activity over past 30 days and muscle‐strengthening activities), low level (those that did not meet the above high‐level conditions were all low levels). Age of HF was defined based on age when you were first told you had congestive heart failure (CHF). Heart disease was diagnosed based on the following conditions: the doctor told you had angina/angina pectoris or the doctor told you had a heart attack (also called myocardial infarction) or doctor or other health professional ever told you that you had coronary heart disease. Stroke was diagnosed based on the doctor or other health professional ever told you had a stroke. Hypertension was diagnosed based on the following conditions: systolic blood pressure (SBP) ≥ 130 mmHg or diastolic blood pressure (DBP) ≥ 80 mmHg or the doctor told you to have hypertension, or taking blood pressure medication. Dyslipidemia was defined as any of the following conditions: total cholesterol (TC) ≥ 200 mg/dL (5.2 mmol/L) or triglyceride (TG) ≥ 150 mg/dL (1.7 mmol/L) or low‐density lipoprotein cholesterol (LDL‐C) ≥ 130 mg/dL (3.4 mmol/L) or high‐density lipoprotein cholesterol (HDL‐C) ≤ 40 mg/dL (1.0 mmol/L) or self‐reported physician diagnosis or taking drug for cholesterol or taking lipid‐lowering medications. Diabetes was defined as any of the following conditions: glycosylated hemoglobin level (HbA1c) ≥ 6.5% or fasting glucose ≥ 126 mg/dL or 2‐h OGTT blood glucose ≥ 200 or self‐reported physician diagnosis or use of insulin or hypoglycemic drugs. CKD was diagnosed based on estimated glomerular filtration rate (eGFR) < 60 mL/min/1.73 m^2^. The eGFR was calculated according to the CKD epidemiology collaboration creatinine equation [18]. Drugs for CVD included primary drugs and secondary drugs. Related drugs that affect blood cell levels included anticoagulants and antiplatelet agents.

### 2.4 Outcomes

Mortality follow‐up was based on data linked to the National Death Index through December 31, 2019 (https://www.cdc.gov/nchs/nhanes/index.htm). The outcomes were all‐cause mortality and CVD mortality. All‐cause mortality was defined as the total number of deaths due to any causes during a given period. CVD mortality was defined as the total number of deaths from heart and cerebrovascular diseases. The median follow‐up time was 107 (48, 157) months.

### 2.5 Statistical Analysis

All data were weighted with SDMVPSU, SDMVSTRA, WTMEC4YR, and WTMEC2YR as weighting variables. Continuous variables were described as the mean ± standard error (mean ± SE), and Student *t* test was used to test the differences between the groups. Categorical variables were represented as count and percentage *n* (%), and *χ* ^2^ test was used for comparison between groups.

The missing values of included variables were handled using the random forest analysis. Sensitivity analyses were performed on the data before and after imputation (Supporting Information S1: Table S1). Covariates were screened via the step‐based regression method in the weighted multivariate Cox proportional hazards analysis (Supporting Information S1: Table S2). Weighted univariate and multivariate Cox models were utilized to explore the effect of PhenoAge and PhenoAgeAccel on all‐cause mortality and CVD mortality in HF patients, with hazard ratios (HRs) and 95% confidence intervals (CIs). Model 1 and Model Ⅰ were weighted univariate Cox models. Model 2 adjusted for age, smoking, diabetes, CKD, overweight, hemoglobin, and anticoagulants. Model II adjusted for age, CKD, drug for CVD, and anticoagulant. To further explore the association in different subgroups of gender, stroke, diabetes, dyslipidemia, and CKD.

Data processing and statistical analyses were performed using SAS 9.4 (SAS Institute Inc., Cary, NC, USA) and R version 4.2.3 (Institute for Statistics and Mathematics, Vienna, Austria). The *p* < 0.05 was considered statistically significant.

## 3 Results

### 3.1 Baseline Information of the Study Population

**Figure 1:**
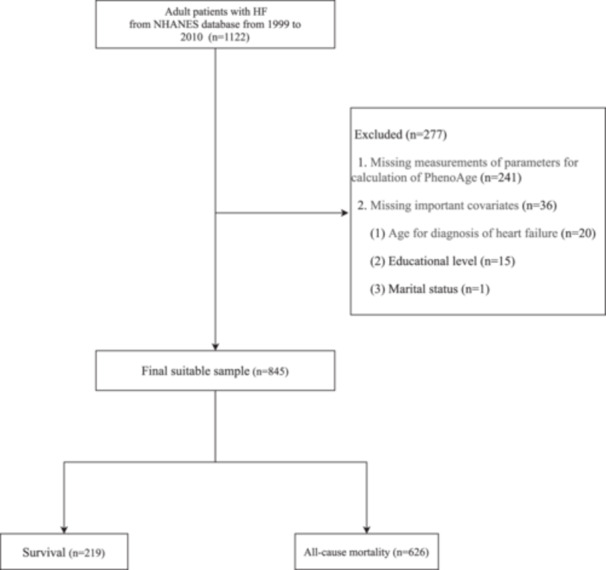
The screen process of the participants analyzed in our study.

According to the inclusion and exclusion criteria, 845 adult Patients with HF admitted to the ICU were finally extracted from the NHANES database (Figure 1). Table 1 provides the characteristics of HF patients stratified according to whether all‐cause death occurred, including 626 all‐cause mortality patients. All‐cause mortality patients were older, more likely to be non‐Hispanic white, unmarried, rarely physically active, overweight, and older for diagnosis of HF; tended to have higher prevalence rates of hypertension, diabetes, and CKD. Those who all‐cause died exhibited a significantly higher uric acid, lower hemoglobin, and higher demand for CVD drugs and anticoagulants. Notably, patients who all‐cause died had an older mean PhenoAge (76.64 vs. 57.75 years) and a high PhenoAgeAccel (1.72 vs. −4.27).

**Table 1:** Baseline and clinical data of patients.

		All‐cause mortality			
Variables	Total (*n* = 845)	No (*n* = 219)	Yes (*n* = 626)	Statistics	*p*
Age, years, mean (SE)	66.51 (0.63)	57.81 (1.21)	70.51 (0.56)	*t* = 0.001	**< 0.001**
Gender, *n* (%)				*χ* ^2^ = 0.385	0.535
Female	345 (43.68)	97 (45.51)	248 (42.83)		
Male	500 (56.32)	122 (54.49)	378 (57.17)		
Race, *n* (%)				*χ* ^2^ = 9.840	**0.043**
Non‐Hispanic White	506 (76.97)	107 (70.08)	399 (80.14)		
Other Hispanic	38 (2.48)	17 (3.35)	21 (2.08)		
Mexican American	104 (3.15)	27 (4.06)	77 (2.74)		
Non‐Hispanic Black	174 (12.79)	59 (16.75)	115 (10.96)		
Other race—including multiracial	23 (4.61)	9 (5.76)	14 (4.08)		
Education level, *n* (%)				*χ* ^2^ = 8.336	0.080
Less than 9th grade	194 (14.32)	37 (8.73)	157 (16.90)		
9–11th grade (includes 12th grade with no diploma)	191 (20.97)	47 (18.89)	144 (21.93)		
High school Grad/GED or equivalent	187 (25.90)	49 (25.55)	138 (26.06)		
Some college or AA degree	188 (26.33)	62 (32.38)	126 (23.55)		
College graduate or above	85 (12.47)	24 (14.45)	61 (11.56)		
PIR, *n* (%)				*χ* ^2^ = 0.158	0.924
< 1	170 (16.23)	50 (17.14)	120 (15.81)		
≥ 1	604 (76.57)	151 (76.17)	453 (76.76)		
Unknown	71 (7.20)	18 (6.70)	53 (7.44)		
Marital status, *n* (%)				*χ* ^2^ = 7.191	**0.007**
Married	420 (52.61)	124 (61.13)	296 (48.69)		
No married	425 (47.39)	95 (38.87)	330 (51.31)		
Smoking, *n* (%)				*χ* ^2^ = 0.831	0.362
No	314 (36.55)	84 (39.20)	230 (35.33)		
Yes	531 (63.45)	135 (60.80)	396 (64.67)		
Alcohol drinking, *n* (%)				*χ* ^2^ = 3.817	0.148
No	233 (31.48)	60 (28.33)	173 (32.93)		
Yes	427 (57.17)	128 (63.37)	299 (54.31)		
Unknown	185 (11.36)	31 (8.30)	154 (12.76)		
Physical activity, *n* (%)				*χ* ^2^ = 11.796	**< 0.001**
Low level	801 (92.96)	194 (86.94)	607 (95.73)		
High level	44 (7.04)	25 (13.06)	19 (4.27)		
Energy intake, mean (SE)	1766.35 (40.68)	1871.62 (72.83)	1717.87 (45.21)	*t* = 1.87	0.064
Age for diagnosis of heart failure, years, mean (SE)	57.13 (0.81)	48.18 (1.54)	61.25 (0.89)	*t* = 0.001	**< 0.001**
Heart disease, *n* (%)				*χ* ^2^ = 1.455	0.228
No	306 (38.50)	87 (42.69)	219 (36.57)		
Yes	539 (61.50)	132 (57.31)	407 (63.43)		
Stroke, *n* (%)				*χ* ^2^ = 3.117	0.077
No	677 (80.30)	190 (85.33)	487 (77.99)		
Yes	168 (19.70)	29 (14.67)	139 (22.01)		
Hypertension, *n* (%)				*χ* ^2^ = 5.226	**0.022**
No	37 (6.20)	17 (10.09)	20 (4.41)		
Yes	808 (93.80)	202 (89.91)	606 (95.59)		
Diabetes, *n* (%)				*χ* ^2^ = 9.434	**0.002**
No	468 (55.60)	138 (66.16)	330 (50.74)		
Yes	377 (44.40)	81 (33.84)	296 (49.26)		
Dyslipidemia, *n* (%)				*χ* ^2^ = 0.068	0.794
No	109 (11.49)	23 (10.92)	86 (11.76)		
Yes	736 (88.51)	196 (89.08)	540 (88.24)		
CKD, *n* (%)				*χ* ^2^ = 35.990	**< 0.001**
No	490 (61.37)	179 (83.36)	311 (51.24)		
Yes	355 (38.63)	40 (16.65)	315 (48.76)		
Overweight, *n* (%)				*χ* ^2^ = 11.607	**0.003**
No	159 (17.68)	29 (13.67)	130 (19.53)		
Yes	633 (77.60)	185 (85.16)	448 (74.12)		
Unknown	53 (4.72)	5 (1.17)	48 (6.35)		
Hemoglobin, g/dL, mean (SE)	13.88 (0.10)	14.33 (0.16)	13.67 (0.09)	*t* = 4.730	**< 0.001**
Uric acid, mg/dL, mean (SE)	6.47 (0.08)	6.10 (0.12)	6.64 (0.11)	*t* = 0.001	**0.002**
Phenotypic age, years, mean (SE)	70.68 (0.91)	57.75 (1.44)	76.64 (0.93)	*t* = 0.001	**< 0.001**
Phenotypic age acceleration, mean (SE)	−0.17 (0.49)	−4.27 (0.75)	1.72 (0.65)	*t* = 0.001	**< 0.001**
Phenotypic age acceleration, *n* (%)				*χ* ^2^ = 31.283	**< 0.001**
< 0	506 (61.00)	171 (79.35)	335 (52.55)		
≥ 0	339 (39.00)	48 (20.66)	291 (47.45)		
Drug for CVD, *n* (%)				*χ* ^2^ = 23.139	**< 0.001**
No	384 (47.91)	125 (63.83)	259 (40.57)		
Yes	461 (52.09)	94 (36.17)	367 (59.43)		
Anticoagulants, *n* (%)				*χ* ^2^ = 8.203	**0.004**
No	697 (82.46)	194 (88.44)	503 (79.70)		
Yes	148 (17.54)	25 (11.56)	123 (20.30)		
Antiplatelet agent, *n* (%)				*χ* ^2^ = 1.626	0.202
No	712 (83.61)	185 (86.35)	527 (82.34)		
Yes	133 (16.40)	34 (13.65)	99 (17.66)		

*Note:* Bold values indicated statistical significance.

Abbreviations: *χ* ^2^: Chi‐square test; CKD: chronic kidney disease; CVD: cardiovascular disease; PIR: family income ratio; SE: standard error; t: *t* test.

### 3.2 Association of PhenoAge and PhenoAgeAccel With the Risk of Mortality

**Figure 2:**
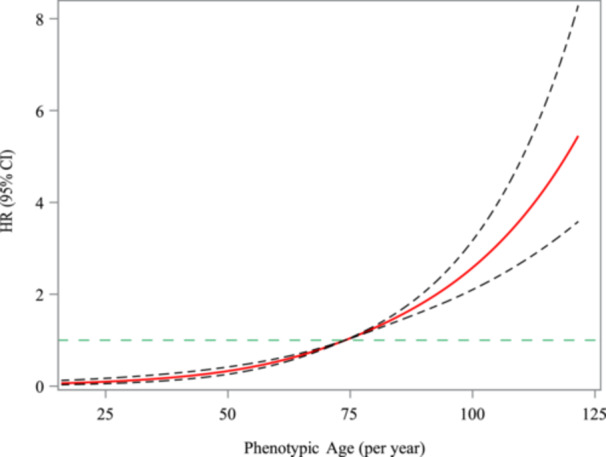
RCS curve of the association between PhenoAge and the risk of mortality in patients with HF.

**Figure 3:**
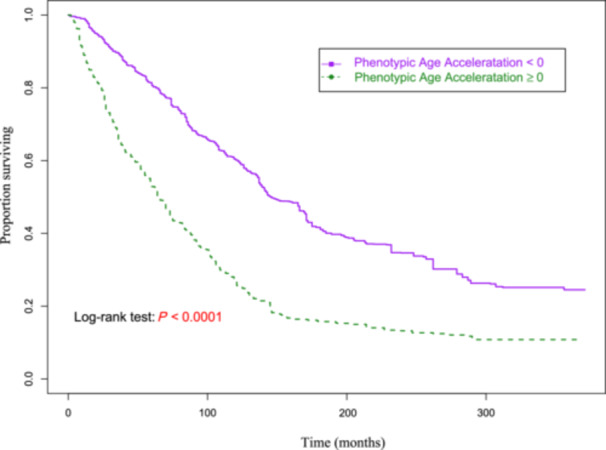
The Kaplan–Meier curve showing the survival probability of patients in different PhenoAgeAccel groups.

To analyze the effect of PhenoAge, PhenoAgeAccel on the risk of mortality in HF patients, weighted univariable and multivariable Cox models were established, respectively. The confounding factors were adjusted in the multivariable Cox model based on the statistically significant variables in Supporting Information S1: Table S2 by step‐based regression. The multivariable Cox model was finally adjusted for all confounding factors, including age, smoking, alcohol drinking, diabetes, CKD, overweight, hemoglobin, drug for CVD, anticoagulants. Table 2 showed that each 1‐ and 10‐year increase in PhenoAge was associated with a 3% (HR = 1.03, 95% CI: 1.03–1.04) and 41% (HR = 1.41, 95% CI: 1.29–1.54) increased risk of all‐cause mortality, respectively. Restricted cubic spline (RCS) curves of PhenoAge‐all‐cause mortality showed that all‐cause mortality risk increased with PhenoAge, when PhenoAge > 75 (per year) (Figure 2). Moreover, when the PhenoAgeAccel < 0 as reference, the ≥ 0 group was associated with a higher risk of all‐cause mortality (HR = 1.91, 95% CI = 1.49–2.45), as shown in Table 2 and Figure 3.

**Table 2:** Association of PhenoAge and PhenoAgeAccel with all‐cause mortality.

	Univariable model		Multivariable model	
Variables	HR (95% CI)	*p*	HR (95% CI)	*p*
Phenotypic age (per year)	1.05 (1.04–1.05)	< **0.001**	1.03 (1.03–1.04)	< **0.001**
Phenotypic age (per 10 years)	1.57 (1.46–1.69)	< **0.001**	1.41 (1.29–1.54)	< **0.001**
Phenotypic age acceleration				
< 0	Ref		Ref	
≥ 0	2.33 (1.85–2.94)	< **0.001**	1.91 (1.49–2.45)	<** 0.001**

*Note:* Univariable model: weighted univariable Cox model; Multivariable model: weighted multivariable Cox model adjusted for all characteristics: age, smoking, alcohol drinking, diabetes, CKD, overweight, hemoglobin, drug for CVD, and anticoagulants. Bold values indicated statistical significance.

Abbreviations*:* CI: confidence interval; HR: hazard ratio; Ref: reference.

### 3.3 Associations of PhenoAge and PhenoAgeAccel With the Risk of Mortality Among Subgroups of Patients With HF

To further explore the association, subgroup analyses stratified by gender and comorbidities (stroke, diabetes, dyslipidemia, and CKD) were performed. Supporting Information S2: Figure 1 and Supporting Information S1: Table S3 showed that older PhenoAge was associated with an increased risk of all‐cause mortality in all subgroups. Supporting Information S3: Figure 2 and Supporting Information S1: Table S4 showed that when the PhenoAgeAccel < 0 as a reference, PhenoAgeAccel ≥ 0 was associated with a higher risk of all‐cause mortality in all subgroups.

## 4 Discussion

To our knowledge, the present study is the first to investigate the effect of PhenoAge and PhenoAgeAccel on mortality risk in patients with HF. The main findings of this study were as follows: (1) older PhenoAge is associated with an increased risk of all‐cause mortality in patients with HF; (2) when the PhenoAgeAccel < 0 as reference, the ≥ 0 group was associated with a higher risk of all‐cause mortality in patients with HF; (3) Subgroup analyses showed that older PhenoAge and PhenoAgeAccel ≥ 0 was associated with higher risk of all‐cause mortality in all subgroups.

Aging is a critical risk factor for most chronic noncommunicable diseases (NCDs) including HF, which is a leading cause of death worldwide [19, 20]. Because of the increasing burden of global population aging, the identification of indicators associated with risk the risk of mortality in patients with HF may help delay premature death and its progression. Currently, many risk factors, including CA, show promise in assessing HF mortality risk [21, 22, 23, 24, 25]. However, aging in humans is highly variable, marking the process of decline in the body's homeostatic capacity, and health status varies greatly at a given CA [26].

Biological age refers to the underlying process that alters the susceptibility to the development of age‐associated diseases, functional impairment, and disabilities, and was superior to predictive power for disease mortality than CA [27, 28]. To estimate biomarkers independent of chronological aging, researchers have developed various BA measures, such as DNA methylation age, telomere length, klotho concentration, and so on [17, 29, 30]. Several studies have shown that epigenetic age acceleration was significantly associated with increased risk of mortality [31, 32, 33], frailty [34], and reduced cognitive performance [35, 36]. Telomere length is considered to as an indicator of cellular aging and is inversely associated with cardiovascular diseases, including hypertension, CAD, and HF [37, 38, 39]. Klotho concentration, as an aging‐related molecule, has been reported to be significantly associated with diabetes all‐cause mortality, cancer mortality and CVD [30, 40]. Recently, investigators used easily accessible clinical tools to develop biological age estimations, such as PhenoAge and PhenoAgeAccel.

PhenoAge was calculated based on a linear combination of CA and nine multisystem clinical chemical biomarkers, has been used as a useful tool for evaluating the effectiveness of interventions [41]. Previous studies have found that generalizability of PhenoAge can facilitate the identification of individuals at different risk of disease and mortality in assessing the risk of various aging outcomes [9, 27]. A recent study has shown that PhenoAge is associated with mortality risk in patients with multivessel coronary artery disease, with a 51% increase in all‐cause mortality risk for each 10‐year increase in PhenoAge [14]. Consistent with this, our findings suggest that each 1‐year and 10‐year increase in PhenoAge were associated with a 3% and 41% increased all‐cause mortality risk, respectively. RCS curves of PhenoAge‐all‐cause mortality showed that all‐cause mortality risk increased with PhenoAge, when PhenoAge > 75 (per year). Subgroup analyses showed that older PhenoAge was associated with an increased risk of all‐cause mortality stratified by gender and comorbidities (stroke, diabetes, dyslipidemia, and CKD) among HF patients. In addition, we found that PhenoAge remained a useful predictor of all‐cause mortality after accounting for CA, that is, PhenoAgeAccel ≥ 0 was associated with a higher risk of all‐cause mortality in HF patients. PhenoAgeAccel, a new measure of BA, represents an aging process characterized by influences on complex biological mechanisms. Ma et al. reported that after adjusting for CA, PhenoAgeAccel was significantly associated with all‐cause mortality in patients with multivessel coronary artery disease [14]. Chen and colleagues have found a positive and linear association of PhenoAgeAccel with all‐cause mortality, and stratified analysis by age and sex found that the association between PhenoAgeAccel and mortality was more significant in the elderly and female subgroups [40]. Based on these conclusions, we stratified by gender and comorbidities (stroke, diabetes, dyslipidemia, and CKD), showing that PhenoAgeAccel ≥ 0 was associated with higher risk of all‐cause mortality in all subgroups.

Multiple underlying mechanisms may explain the pathophysiological mechanisms of association between PhenoAge, PhenoAgeAccel, and mortality risk in HF patients. First, with aging, there is a stable cell cycle arrest tendency, called cellular senescence, long‐term accumulation of senescent cells will lead to cardiac function decline [42, 43]. Second, aging can lead to chronic inflammation, including immune system dysregulation, oxidative stress, chronic infections, and so on [44]. Inflammation is thought to be highly sensitive to BA, frailty, cardiovascular disease, and premature death [44, 45, 46]. Third, aging affects all organ systems, resulting in impaired reserve capacity and reduced ability to respond to stress. Although there are organ‐differential and organ‐specific resilience and fragility in aging, frailty refers to the cumulative decline and increased homeostasis imbalance that precedes the ultimate consequence of aging: death [47].

Our findings have clinical implications for patients with HF. While PhenoAge and PhenoAgeAccel cannot replace a well‐established HF risk prediction system, they can serve as useful tool to facilitate the identification of individuals at risk and the evaluation of the efficacy of HF treatment. The ability of PhenoAge to capture indicators of preclinical aging and future morbidity/mortality risk could facilitate the evaluation of intervention effects while avoiding the need for decades of follow‐up [48]. PhenoAgeAccel is also a marker that could be used to monitor the aging process before disease symptoms appear [9]. It is well known that changes in aging begin long before disease occurs [49], so interventions to slow aging would be most effective in reducing disease incidence and mortality if identified early in the life course before significant accumulation of aging‐related damage. Physicians can better understand the biological status of HF patients by assessing changes in PhenoAge and PhenoAgeAccel. This provides the basis for individualized treatment and intervention. Those patients with higher PhenoAge and PhenoAgeAccel, need to be treated and monitored more aggressively to control the progression of HF and reduce the risk of mortality.

The present study has several strengths. First, this study is the first to explore the association of PhenoAge and PhenoAgeAccel with the risk of mortality in patients with HF. Second, the indicators of PhenoAge are easy to detect, which provides a convenient tool for long‐term prognosis risk monitoring in patients with HF. There are also limitations. First, this study is a retrospective cohort study, which inevitably has a certain selection bias. Second, the sample of our study was limited, but the follow‐up duration was sufficient to ensure that the number of samples with outcome events occurred. Third, there is a lack of longitudinal data for PhenoAge in the database. The association of PhenoAge change and rate of change with the risk of mortality in patients with HF needs to be further studied.

## 5 Conclusion

Our study shows that after adjusting for CA, older PhenoAge was associated with an increased risk of all‐cause mortality in HF patients. PhenoAge and PhenoAgeAccel can be used as convenient tools to facilitate identification at‐risk individuals of HF and evaluation of intervention efficacy.

## Conflicts of Interest

The authors declare no conflicts of interest.

## Supporting information

Supporting information.

Supplementary Figure 1 Forest plot revealing the associations of PhenoAge with the risk of mortality among subgroups of patients with HF.

Supplementary Figure 2 Forest plot revealing the associations of PhenoAgeAccel with the risk of mortality among subgroups of patients with HF.

## Data Availability

The datasets generated and/or analyzed during the current study are available in the NHANES database, https://wwwn.cdc.gov/nchs/nhanes/.
