# Supplementary material for: Association Between Phenotypic Age and the Risk of Mortality in Patients With Heart Failure: A Retrospective Cohort Study
Source: Clin Cardiol. 2024 Aug 8;47(8):e24321. doi: 10.1002/clc.24321 (PMC11307102; doi:10.1002/clc.24321)
Supplement: Supplementary file 1 — Supporting information. [file CLC-47-e24321-s002.docx]

Supplemental Table 1 Sensitivity analysis.

| **Variables** | **Before imputation (n=845)** | **After imputation (n=845)** | **Statistics** | ***P*** |
| --- | --- | --- | --- | --- |
| Energy intake, kcal, Mean (S.E) | 1767.72 (41.79) | 1766.35 (40.68) | t=0.001 | 0.795 |

Note: T: T-test; S.E: standard error.

Supplemental Table 2 Results of covariate screening.

|  | **All-cause mortality** | |
| --- | --- | --- |
| **Variables** | **HR (95%CI)** | ***P*** |
| Age | 1.05 (1.04-1.06) | **<.001** |
| Gender |  |  |
| Female | Ref |  |
| Male | 1.03 (0.86-1.24) | 0.731 |
| Race |  |  |
| Non-Hispanic White | Ref |  |
| Non-Hispanic Black | 0.66 (0.38-1.12) | 0.125 |
| Mexican American | 0.73 (0.46-1.14) | 0.166 |
| Other Hispanic | 0.69 (0.54-0.87) | **0.002** |
| Other Race - Including Multi-Racial | 0.81 (0.44-1.47) | 0.484 |
| Education level |  |  |
| Less Than 9th Grade | Ref |  |
| 9-11th Grade (Includes 12th grade with no diploma) | 0.84 (0.61-1.16) | 0.285 |
| High School Grad/GED or Equivalent | 0.87 (0.64-1.19) | 0.397 |
| Some College or AA degree | 0.67 (0.49-0.93) | **0.015** |
| College Graduate or above | 0.68 (0.47-0.99) | **0.045** |
| PIR |  |  |
| <1 | Ref |  |
| >=1 | 1.05 (0.77-1.43) | 0.749 |
| Unknown | 1.29 (0.82-2.02) | 0.275 |
| Marital status |  |  |
| Married | Ref |  |
| No married | 1.31 (1.08-1.58) | **0.005** |
| Smoking |  |  |
| No | Ref |  |
| Yes | 1.02 (0.82-1.27) | 0.860 |
| Alcohol drinking |  |  |
| No | Ref |  |
| Yes | 0.81 (0.61-1.07) | 0.133 |
| Unknown | 1.07 (0.83-1.39) | 0.590 |
| Physical activity |  |  |
| Low level | Ref |  |
| High level | 0.49 (0.28-0.84) | **0.010** |
| Energy intake | 1.00 (1.00-1.00) | 0.060 |
| Heart disease |  |  |
| No | Ref |  |
| Yes | 1.03 (0.82-1.29) | 0.794 |
| Stroke |  |  |
| No | Ref |  |
| Yes | 1.32 (1.03-1.69) | **0.027** |
| Hypertension |  |  |
| No | Ref |  |
| Yes | 1.86 (1.03-3.36) | **0.039** |
| Diabetes |  |  |
| No | Ref |  |
| Yes | 1.50 (1.19-1.89) | **<.001** |
| Dyslipidemia |  |  |
| No | Ref |  |
| Yes | 0.88 (0.62-1.25) | 0.471 |
| CKD |  |  |
| No | Ref |  |
| Yes | 2.49 (1.99-3.11) | **<.001** |
| Overweight |  |  |
| No | Ref |  |
| Yes | 0.81 (0.62-1.05) | 0.114 |
| Unknown | 1.84 (1.12-3.02) | **0.015** |
| Hemoglobin | 0.82 (0.78-0.88) | **<.001** |
| Uric acid | 1.15 (1.08-1.22) | **<.001** |
| Drug for CVD |  |  |
| No | Ref |  |
| Yes | 1.79 (1.45-2.23) | **<.001** |
| Anticoagulants |  |  |
| No | Ref |  |
| Yes | 1.63 (1.30-2.03) | **<.001** |
| Antiplatelet agent |  |  |
| No | Ref |  |
| Yes | 1.17 (0.92-1.49) | 0.192 |

Note: Ref: Reference; HR: Hazard Ratio; CI: Confidence Interval; PIR: family income ratio; CKD: chronic kidney disease; CVD: cardiovascular disease.

Supplementary Table 3 Associations between PhenoAge (per 10 years) and all-cause mortality among subgroups of patients with HF.

|  | **All-cause mortality** | |
| --- | --- | --- |
| **Variables** | **HR (95%CI)** | ***P*** |
| Gender |  |  |
| Male (n=500) | 1.31 (1.14-1.50) | **<.001** |
| Female (n=345) | 1.52 (1.26-1.82) | **<.001** |
| Stroke |  |  |
| No (n=677) | 1.37 (1.23-1.54) | **<.001** |
| Yes (n=168) | 1.57 (1.20-2.06) | **<.001** |
| Diabetes |  |  |
| No (n=468) | 1.49 (1.26-1.76) | **<.001** |
| Yes (n=377) | 1.27 (1.09-1.49) | **<.001** |
| Dyslipidemia |  |  |
| No (n=109) | 1.62 (1.25-2.10) | **<.001** |
| Yes (n=736) | 1.38 (1.22-1.55) | **0.003** |
| CKD |  |  |
| No (n=490) | 1.37 (1.15-1.64) | **<.001** |
| Yes (n=355) | 1.40 (1.22-1.60) | **<.001** |

Note: Ref: Reference; HR: Hazard Ratio; CI: Confidence Interval; CKD: chronic kidney disease.

Supplementary Table 4 Associations between PhenoAgeAccel and all-cause mortality among subgroups of patients with HF.

|  | **Phenotypic age acceleration** |  | **All-cause mortality** | |
| --- | --- | --- | --- | --- |
| **Variables** |  | **n (%)** | **HR (95%CI)** | ***P*** |
| Gender |  |  |  |  |
| Male | <0 | 286 (59.50) | Ref |  |
|  | ≥0 | 214 (40.50) | 1.88 (1.37-2.57) | **<.001** |
| Female | <0 | 220 (62.93) | Ref |  |
|  | ≥0 | 125 (37.07) | 1.98 (1.23-3.19) | **0.005** |
| Stroke |  |  |  |  |
| No | <0 | 410 (62.39) | Ref |  |
|  | ≥0 | 267 (37.61) | 2.05 (1.50-2.81) | **<.001** |
| Yes | <0 | 96 (55.32) | Ref |  |
|  | ≥0 | 72 (44.68) | 2.03 (1.25-3.29) | **0.004** |
| Diabetes |  |  |  |  |
| No | <0 | 365 (80.97) | Ref |  |
|  | ≥0 | 103 (19.03) | 2.18 (1.50-3.17) | **<.001** |
| Yes | <0 | 141 (35.99) | Ref |  |
|  | ≥0 | 236 (64.01) | 1.60 (1.08-2.39) | **0.020** |
| Dyslipidemia |  |  |  |  |
| No | <0 | 60 (56.07) | Ref |  |
|  | ≥0 | 49 (43.93) | 3.08 (1.72-5.54) | **<.001** |
| Yes | <0 | 446 (61.64) | Ref |  |
|  | ≥0 | 290 (38.36) | 1.77 (1.30-2.42) | **<.001** |
| CKD |  |  |  |  |
| No | <0 | 358 (73.09) | Ref |  |
|  | ≥0 | 132 (26.91) | 2.26 (1.49-3.42) | **<.001** |
| Yes | <0 | 148 (41.79) | Ref |  |
|  | ≥0 | 207 (58.21) | 1.68 (1.20-2.37) | **0.003** |

Note: Ref: Reference; HR: Hazard Ratio; CI: Confidence Interval; CKD: chronic kidney disease.
