# Supplementary figures and images for: Association Between Phenotypic Age and the Risk of Mortality in Patients With Heart Failure: A Retrospective Cohort Study
Source: Clin Cardiol. 2024 Aug 8;47(8):e24321. doi: 10.1002/clc.24321 (PMC11307102; doi:10.1002/clc.24321)

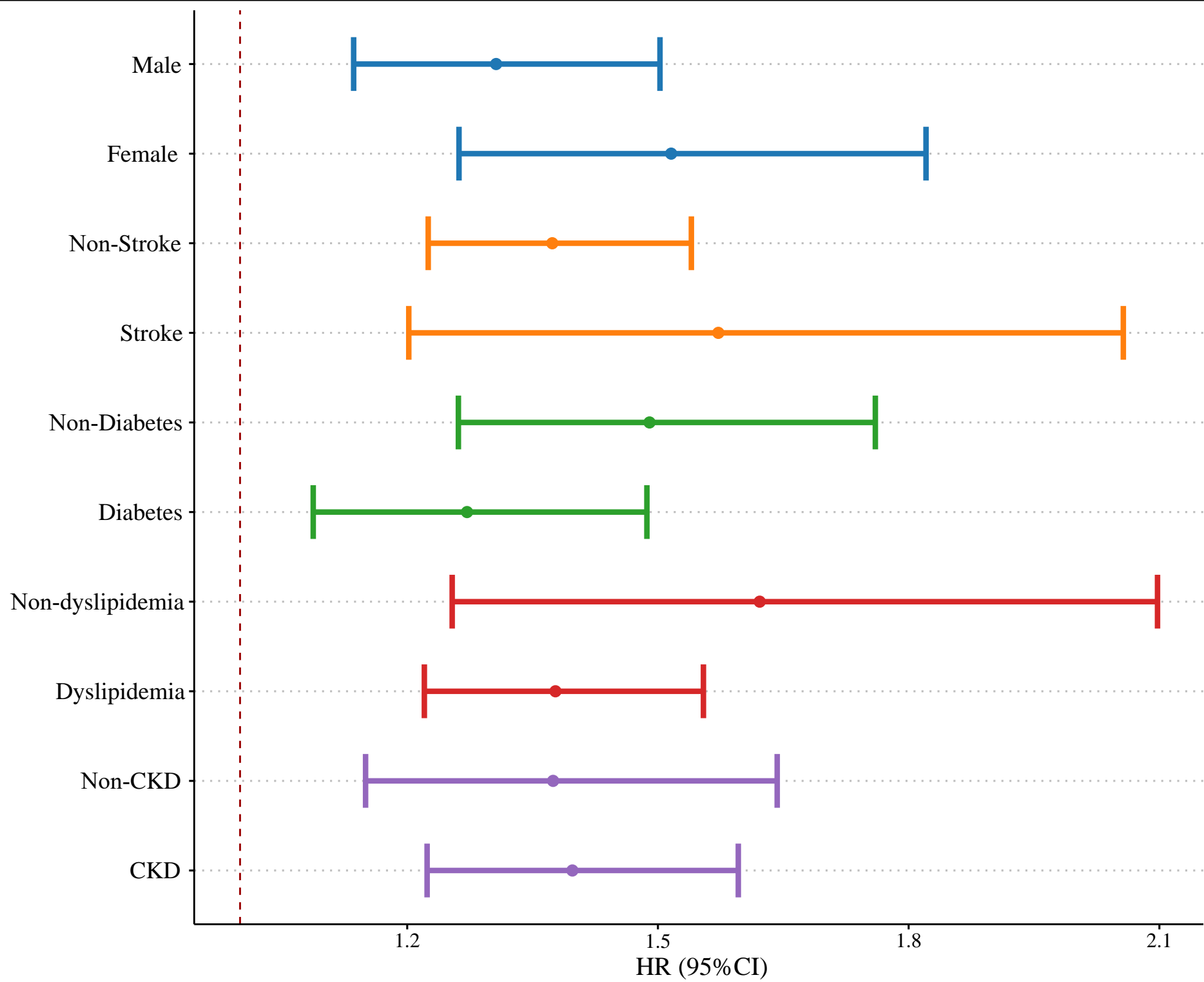

Supplement: Supplementary file 2 — Supplementary Figure 1 Forest plot revealing the associations of PhenoAge with the risk of mortality among subgroups of patients with HF. [file CLC-47-e24321-s001.pdf]

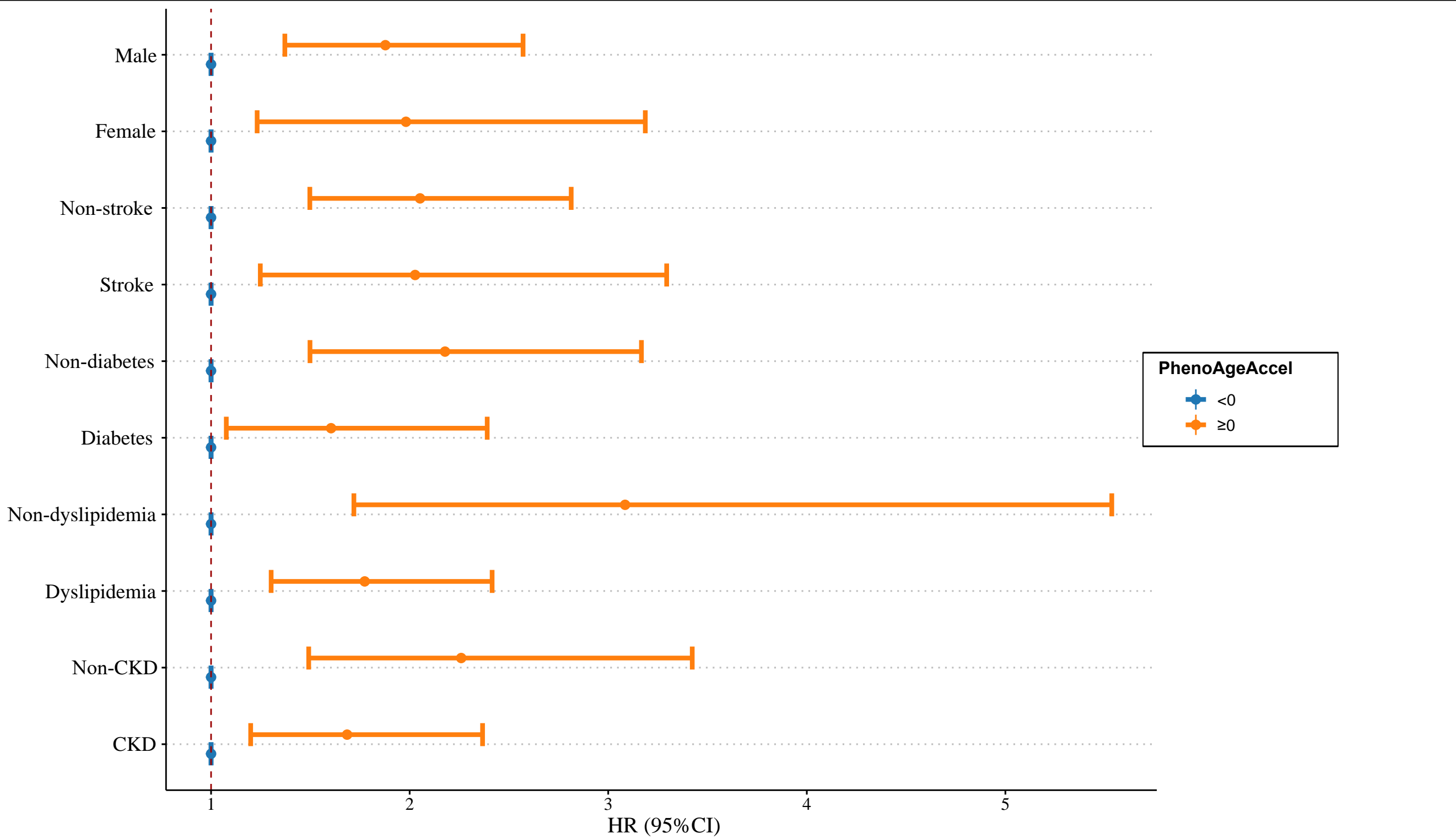

Supplement: Supplementary file 3 — Supplementary Figure 2 Forest plot revealing the associations of PhenoAgeAccel with the risk of mortality among subgroups of patients with HF. [file CLC-47-e24321-s003.pdf]
